# Development of Genetic Testing for Fragile X Syndrome and Associated Disorders, and Estimates of the Prevalence of *FMR1* Expansion Mutations

**DOI:** 10.3390/genes7120110

**Published:** 2016-11-30

**Authors:** James N. Macpherson, Anna Murray

**Affiliations:** 1 Wessex Regional Genetics Laboratory, Salisbury NHS Foundation Trust, Salisbury District Hospital, Salisbury SP2 8BJ, UK; 2 Medical School, University of Exeter, RILD Level 3, Royal Devon & Exeter Hospital, Barrack Road, Exeter EX2 5DW, UK; A.Murray@exeter.ac.uk

**Keywords:** Fragile X, prevalence, dynamic mutation

## Abstract

The identification of a trinucleotide (CGG) expansion as the chief mechanism of mutation in Fragile X syndrome in 1991 heralded a new chapter in molecular diagnostic genetics and generated a new perspective on mutational mechanisms in human genetic disease, which rapidly became a central paradigm (“dynamic mutation”) as more and more of the common hereditary neurodevelopmental disorders were ascribed to this novel class of mutation. The progressive expansion of a CGG repeat in the *FMR1* gene from “premutation” to “full mutation” provided an explanation for the “Sherman paradox,” just as similar expansion mechanisms in other genes explained the phenomenon of “anticipation” in their pathogenesis. Later, *FMR1* premutations were unexpectedly found associated with two other distinct phenotypes: primary ovarian insufficiency and tremor-ataxia syndrome. This review will provide a historical perspective on procedures for testing and reporting of Fragile X syndrome and associated disorders, and the population genetics of *FMR1* expansions, including estimates of prevalence and the influence of AGG interspersions on the rate and probability of expansion.

## 1. Introduction and History

Fragile X syndrome (OMIM #300624) owes its name to the cytogenetics phenomenon by which it was both characterized [1] and, for many years, routinely diagnosed, namely, the presence of a rare fragile site at the putative genetic locus [fra(Xq27.3)]. Although it was recognized as the most common of the heritable learning disability syndromes—early estimates of prevalence [2,3] ranging from 1 in 1362 to 1 in 2600 males—efforts to elucidate the candidate gene and its mode of inheritance proved elusive. The phenotype, more severe and well-defined in males than in females, typically featured a long face, large ears, moderate to severe learning disability with some autistic features, and macroorchidism in post-pubertal males [4]; this was invariably accompanied by expression of a folate-sensitive fragile site in up to 50% of cells in males (but not always in females). Its inheritance followed a non-Mendelian, apparently X-linked dominant pattern in which the full syndrome could only be inherited maternally, but the mutation could nevertheless be carried and passed on to daughters by non-penetrant “transmitting” males, with a marked increase in risk of the disease through successive generations known as the “Sherman paradox” [5].

**Figure 1:**
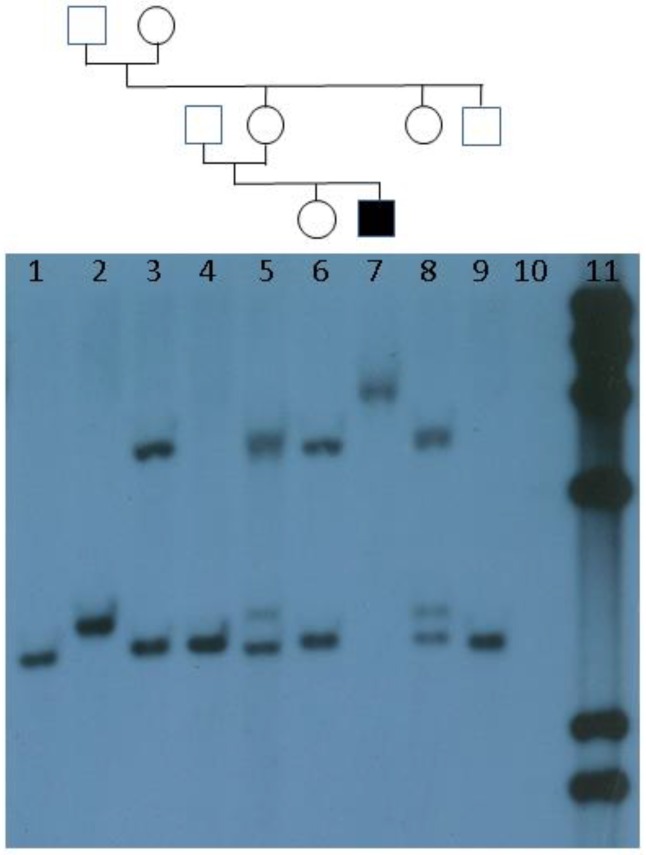
Southern blot of *FMR1* repeat showing inheritance of premutation from normal transmitting male (lane 2) to two daughters (lanes 5 and 8) and expansion to full mutation in grandson (lane 7). Lane 11: λ*Hin*dIII marker.

The incomplete penetrance in males and variable penetrance/expressivity in females, both of the clinical and the cytogenetic phenotype, together with the nonspecific clinical phenotype in children, created an urgent need for a more sensitive molecular test especially with a view to earlier diagnosis in childhood; however, cloning and precise localization of the gene proved difficult. The concept of a “premutation” which could be silently transmitted over several generations into a “full mutation” had been suggested [6] but no convincing molecular basis for it could be envisaged. Finally, in 1991, four independent research groups [7,8,9,10] discovered both the putative gene *(FMR1)* and the mutational mechanism: an expansion of a trinucleotide (CGG) repeat tract at the 5′ end of the gene, a novel phenomenon in genetics. Data from Southern blots using double restriction enzyme digests (Figure 1) showed clearly that the nonpenetrant “transmitting” males had smaller expansions than the Fragile X syndrome males—supporting the premutation hypothesis—and also that the larger expansions in the Fragile X males were hypermethylated, providing a clue to the pathogenesis of the condition: a loss of function of the protein (FMRP), reducing synaptic plasticity in brain and central nervous system (CNS) neurones [11]. In succeeding generations, the size of the expansion progressively and markedly increased until it reached a threshold of approximately 0.6 kb above normal size, above which all expansions were methylated. This “dynamic mutation” [12] process not only explained the reduced penetrance in males but also solved the Sherman paradox [13]. Around 10%–15% of full mutation patients were also found to be mosaic for lower levels of a premutation [14], which correlated with a milder Fragile X phenotype in these individuals [15].

The ability to test directly for the Fragile X mutation by molecular genetics methods revolutionized both the diagnosis of the condition and the accuracy of estimates of its prevalence. Previously, using cytogenetic expression of fra(X) as the sole diagnostic tool, it was not possible to distinguish between the two adjacent fragile sites *FRAXA* and *FRAXE* at the resolution used; hence, Fragile X syndrome was overdiagnosed as a proportion of false “positive” cases were actually expressing *FRAXE* [fra(Xq28)], which is associated with a much milder, non-syndromic learning disability—the gene at this locus, *AFF2*, was later shown to have a 5′ trinucleotide GCC repeat, which could expand in similar fashion [16]. Moreover, females with Fragile X did not always express fra(Xq27.3) so a proportion of female probands remained undiagnosed (false negatives). Hence, the cytogenetic test had to be complemented by linkage analysis, particularly for carrier testing and prenatal diagnosis, but the markers used were typically at least five centimorgan from the gene locus and often of limited informativity. Further elucidation of the *FMR1* gene showed that the CGG repeat tract was polymorphic in the normal population, and led to the development of a PCR test for the precise number of CGG repeats [13]; this in turn allowed the limits of normal, premutation and full mutation to be determined and also revealed the presence of a fourth allele class: those in between the normal and premutation states, which were at increased risk of expansion but had not yet become progressively unstable, the “intermediate” or “grey zone” alleles [17] (see Table 1 for definitions of allele classes). The molecular data also explained why de novo mutations had not been observed, in that all probands were ascertained as full mutations arising from either premutations or full mutations in the preceding generation.

## 2. Population Studies and Prevalence

Prevalence estimates were revised down to around 1 in 4000 males once the existing Fragile X families were re-tested by molecular methods (Southern blot and PCR) [18]. A subsequent screening survey of schoolboys with special educational needs and their mothers [19] further revised the prevalence of *FRAXA* full mutations down to 1 in 5,500 males and of *FRAXE* full mutations down to 1 in 23,000 males, once the bias of ascertainment had been eliminated. These estimates are probably still the best available today for Caucasian populations, although there are some ethnic groups in which a significantly higher prevalence of *FRAXA* has been recorded, notably those of Tunisian Jewish origin [20]. Studies in other ethnic groups are relatively smaller, making it difficult to make accurate estimates of prevalence. A large study of African Americans (N = 1,103) found a higher point estimate for the prevalence of the full mutation, but the confidence intervals overlapped the Caucasian prevalence [21].

The prevalence of premutations and intermediate alleles is harder to determine accurately, as the data published by different researchers do not always use the same size ranges and there is not a universally accepted consensus on the most appropriate size limits for normal and intermediate alleles (Table 1). In the UK, diagnostic laboratories have preferred the traditional definition of a premutation as an allele with definite, irreversible and accelerating instability which will inevitably progress to a full mutation in only a few (typically 1–3) generations; this equates to approximately 59 –200 repeats, although there are rare examples of alleles smaller than 59 repeats showing such rapid expansion [22,23]. Other groups, particularly in the USA, have favoured a broader premutation range of 55–200 repeats, and consequently narrower intermediate range (“grey zone”) of 45–54 repeats.

Our data from large screening surveys in the UK [24] suggest that many alleles in the range 55–58 repeats are transmitted either stably or with only minor instability (either expansion or contraction by one or two repeats), so we believe that these are more accurately classed as intermediate alleles (discussed in more detail in Section 8 below).

## 3. Factors Affecting Stability and Expansion of the CGG/AGG Repeat Tract

**Figure 2:**
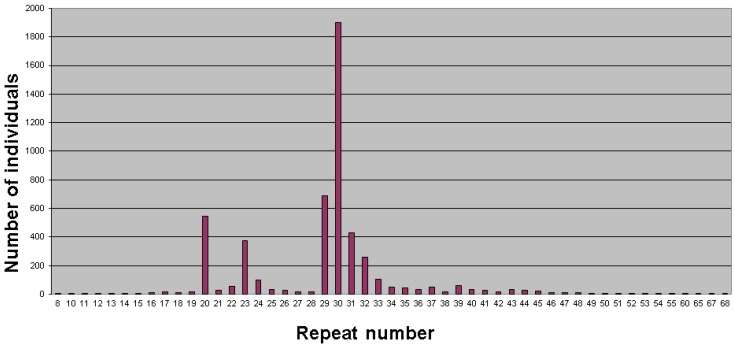
Distribution of CGG repeats in the general population (taken from a screening survey of children in Avon, England).

The range of repeat size alleles in the normal population varies little between most ethnic groups studied; the distribution for a UK population is shown in Figure 2. The frequency of alleles at the upper end of the normal range (40 repeats and above), combined with empirical observations of stability, helps to determine the most suitable upper limit of “normal,” which is usually quoted either as 45 or 46 repeats. There is, however, no absolute and rigid demarcation between normal, intermediate and premutation allelic states, but rather a statistical gradation of probability of expansion which depends not only on size but also upon other factors, such as the number and position of interspersed AGG repeats, the longest uninterrupted CGG tract, and flanking haplotype [24,25,26,27,28,29].

Most repeat tracts in normal and intermediate alleles are not, in fact, pure CGG but have at least two interspersed AGG motifs, the positions of which are semi-conserved at the 5′ end [25,27,28,30,31] and which it is thought act as an anchor to prevent instability due to slippage replication or unequal crossing-over. Haplotype analysis has showed strong linkage disequilibrium between flanking microsatellites and particular repeat size classes, which also correlates with the interspersion pattern: one particular haplotype for three flanking microsatellites (*DXS548*, *FRAXAC1*, *FRAXAC2*: 7-3-4+) is associated with the most common normal allele and AGG pattern (30 repeats, 10+9+9 where + represents an AGG motif after the preceding number of CGG repeats). Another haplotype (2-1-3) is found associated mainly with intermediate alleles as well as with full and premutations. Other, less common haplotypes are associated both with different sizes of normal allele and with full mutations, and thus may indicate a more rapid rate of expansion between normal and premutation without a lasting intermediate stage [32]. There appear to be at least two mechanisms of instability: a slow creeping expansion (“slippage”) typical of intermediate alleles with two AGG interspersions at positions 11 and 21 (10+9+n) and a more pronounced and accelerating expansion typical of pure CGG repeat tracts [26]. This is supported by the fact that most true premutations tested are devoid of AGG interspersions or retain only the 5′ AGG [33] (though this is subject to ascertainment bias, as most premutations come to attention via a proband who already has a full mutation).

The empirical hypothesis, therefore, is that the most common “origin” of a Fragile X mutation is the loss of an AGG interspersion or its conversion to CGG, which increases the length of pure CGG tract, thus making the allele significantly more prone to expansion. This could in theory lead to a normal allele mutating to an intermediate allele or a premutation, though this has not been observed in a single step. Rarely, an “intermediate” allele shows the rapid instability characteristic of a premutation, but in one report a 52-repeat allele progressed to 56 repeats, with concomitant loss of both AGGs, then to a full mutation in only two meioses [23]. The observation that in alleles with only one AGG it is almost always the one in the 5′ position that is preserved (10 or 11 repeats in) may give a clue to the mechanism of AGG loss (such as hairpin-loop or other slipped-strand formation) [34,35]; alternatively, it might suggest that loss of the 5′ AGG first would be the more catastrophic event leading to rapid subsequent loss of all 3′ interspersions and, hence, much more pronounced instability than expected from the initial loss of the 3′ AGG.

The occurrence of several different haplotypes on Fragile X alleles suggests a multiple origin for the Fragile X expansion mutation, but also the association of intermediate alleles with the 2-1-3 haplotype points to a founder effect for the “slow path” mutation on this haplotype [26]. The postulated “rapid path” expansion mechanism is likely more complex and could involve a *cis*-acting modifier of repeat length stability, a candidate for which has been identified 53 kb proximal to the repeat [36,37].

## 4. Current Diagnostic Procedure

**Figure 3:**
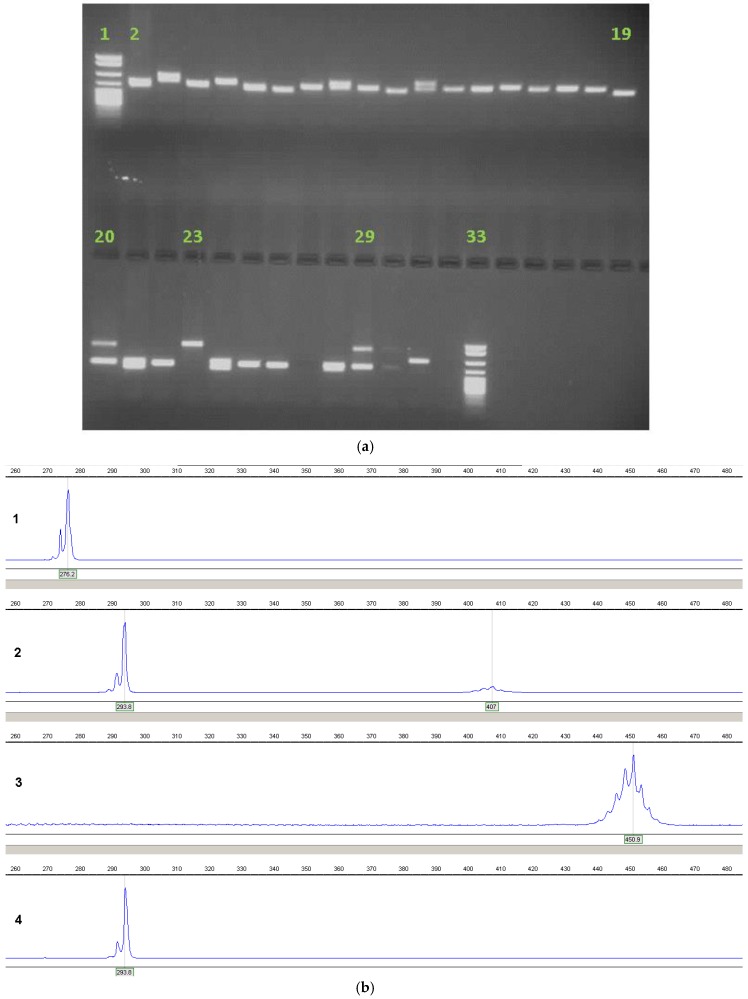
(**a**): *FMR1* PCR run on a 1.7% agarose gel (for method, see reference [38]). Lanes 2–19: Normal alleles; lanes 20, 23, 29: Premutations; lanes 1, 33: pBR322*Msp*I size marker; (**b**): *FMR1* PCR analysed on an ABI3130^TM^ (Applied Biosystems, Foster City, CA, USA) automated sequencer. Panel 1: Male, 23 repeats; Panel 2: Female, 30 and approx. 72 repeats; Panel 3: Male, approx. 89 repeats; Panel 4: Male, 30 repeats.

The vast majority of referrals for *FRAXA* testing to diagnostic laboratories are children between the ages of 2 and 10, before the classical Fragile X phenotype would be manifest, and, hence, their clinical features are typically common conditions (developmental delay, autism, attention-deficit-hyperactivity disorder, speech and language delay, challenging behaviour, etc.) which are not specific to Fragile X syndrome; therefore, an inexpensive method of excluding Fragile X in large patient batches is required, with a high sensitivity. A long-template PCR technique [38] will detect normal, intermediate and most premutation alleles, thereby excluding a diagnosis of Fragile X in all but those who have a large expansion or females who may be homozygous for a normal CGG repeat allele, who will require further analysis either by Southern blot or by using commercial kits such as Amplidex^TM^ (Asuragen, Austin, TX, USA). The PCR products can be visualized on an agarose gel, but analysis on an automated sequencer enables better resolution of the repeat size (Figure 3) in most cases to a confidence limit of +/−1 repeat in the normal range, +/−2 in the intermediate range and +/−3 to 4 in the premutation range. Larger premutations, over 100 repeats, are likely to require second-line analysis both for accurate sizing and to exclude mosaicism for a full mutation.

**Figure 4:**
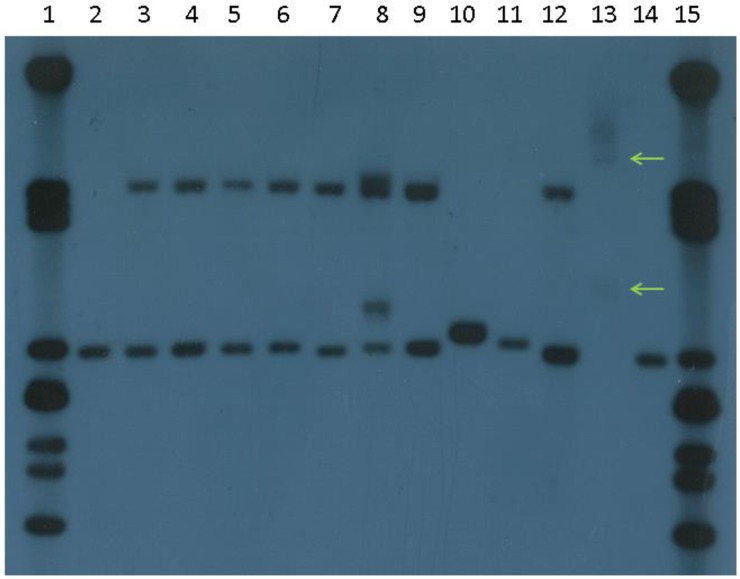
Southern blot of *FMR1* repeat using *Bst*ZI/*Eco*RI digest and StB12.3 probe. Lanes 2 and 14: Normal males; lanes 3–7, 9 and 12: Normal females; Lane 8: Premutation female; Lane 10: Premutation male; Lane 11: Intermediate male; Lane 13: Mosaic male (premutation and full mutation arrowed); Lanes 1 and 15: λ*Pst*I marker.

Southern blot analysis still uses the same basic technology as the original testing when the *FMR1* gene was first identified [14]: a double digest using a standard enzyme such as *Eco*RI and a methylation-sensitive enzyme such as *Eag*I followed by hybridization to a cloned probe homologous to the fragment reveals both the size and methylation status of any expansions (Figure 4). Methylation status is useful not only to distinguish full mutations from large premutations but also to estimate the level of mosaicism, if any. True mosaics have different-sized expansions, one of premutation and one of full mutation size, whereas “methylation mosaics” have apparently one size of expansion which is incompletely methylated. In females, Southern blots can reveal if X-inactivation is skewed either towards the normal or mutant allele; however, despite a weak correlation between the proportion of normal active X and IQ [39], this has not proved useful in predicting the severity of the phenotype in females with a full mutation presumably because it does not reflect the X-inactivation pattern in tissues other than blood. Southern blot hybridization has a good sensitivity of detection of expansions of all sizes, but is labour-intensive with a relatively long turnaround time and does not give a precise repeat size. It is also vulnerable to technical problems with probe labelling efficiency and signal-to-noise ratio, which can require extensive and laborious re-optimization; hence, the need for a rapid, reliable, routine test that can detect all sizes of expansion as well as normal alleles. PCR-based commercial *FMR1* kits are now gradually replacing Southern blotting as the preferred second-line test for diagnosis in patients for whom Fragile X cannot be excluded by standard PCR.

**Figure 5:**
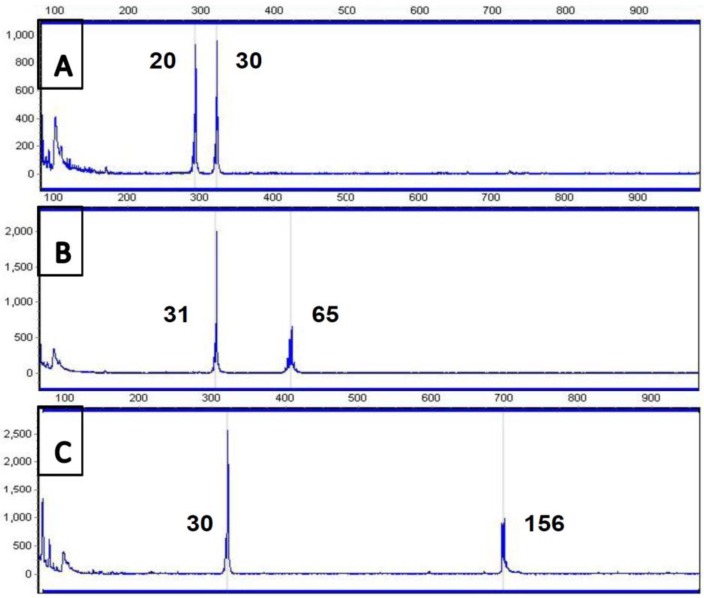
Electropherograms of CGG repeat amplification with Amplidex^TM^ kit in females. Panel **A**: normal alleles (20 and 30 CGG repeats). Panel **B**: mid-sized premutation carrier (31 and 65 CGG repeats). Panel **C**: large premutation carrier (30 and 156 CGG repeats).

The Amplidex^TM^ kit from Asuragen is the most widely used of these products, although several others are also on the market. The method uses a proprietary mastermix that is able to amplify the CG-rich sequence that is often refractory to PCR application and overcomes the issue of preferential amplification of the smaller CGG repeat allele in samples from females (Figure 5). There is also a version of the kit that can be used to determine methylation status and estimation of the number and position of AGG interspersions. Its drawbacks are that of cost (at the time of writing, around 40 GBP per patient) and that the standard kit does not give either size or methylation status within the full mutation range. In some cases, therefore, Southern blot analysis may still be required as a second or even a third-line test, e.g., for prenatal diagnosis (see Section 6 below) or for diagnosis of patients where sizing or determination of the methylation status of an expansion is critical.

The ability to measure the number and position of AGG interspersions would be extremely useful for distinguishing between stable intermediate alleles and those likely to be prone to expansion, and, hence, become premutations, especially in women considering whether to opt for prenatal diagnosis. Several different technologies have been employed for this purpose, though none are straightforward. The most direct method, Sanger sequencing, is hampered by difficulties in amplification of long CG-rich tracts by PCR and may not easily distinguish two very similar repeat profiles of different alleles in a female. The Amplidex^TM^ TP-PCR application gives the approximate position of AGGs from observation of antinodes in the electropherogram, but again these are less evident in a female than in a male. Nevertheless, both these approaches have been utilized successfully [29]. Alternatively, digestion with a restriction enzyme (*Mnl*I) that cuts adjacent to the AGG motif may be used to give a likely approximation of AGG positions [25,28] but the pattern of fragments generated by this method requires careful interpretation, is not appropriate for testing heterozygous females and requires Southern blot analysis. The search for a comprehensive single-step test to detect all repeat sizes, methylation status and AGG interspersion patterns, and, hence, eliminate the need for second-line testing, remains the “Holy Grail” of Fragile X diagnostics. A recently developed diagnostic assay using combinations of PCR, TP-PCR and methylation-specific PCR [40] has made some progress, but is not yet widespread in clinical practice. At present, next-generation sequencing by massively parallel sequencing is not suitable for sizing triplet repeat expansions; however, there is potential for third-generation sequencing methods (long-read or single-molecule sequencing) to be adapted for this purpose [41] and thus provide a universal platform in the future for the diagnosis of Fragile X and other trinucleotide repeat disorders. One of the challenges for single-molecule sequencing of a specific locus such as the *FMR1* CGG repeat is to find a method to enrich the target sequence that does not involve PCR, which is notoriously inefficient at amplifying large repetitive motifs.

## 5. Testing Criteria

The high rate of Fragile X referrals to diagnostic laboratories in the absence of a clinically specific phenotype has created pressure to implement consensus testing criteria to reduce the proportion of blanket “exclusion” testing and thus target resources towards the most likely patients. The UK Genetic Testing Network (UKGTN) published recommended minimum criteria for testing males and females with developmental delay/learning difficulty and for carrier testing in Fragile X families [42] following a meeting in 2008 attended by representatives from clinical geneticists, genetics nurses, clinical laboratory scientists and Fragile X support groups; these criteria are now adopted by most UK regional genetics laboratories. It is also common practice to triage the testing pathway using genome-wide analysis (array-CGH) as the primary test for developmental delay, followed by *FMR1* testing only if the array-CGH does not detect a clinically significant imbalance.

## 6. Prenatal Diagnosis

Prenatal testing for Fragile X is routinely offered to women with a premutation or a full mutation (but not to couples where the man is a premutation carrier, as paternally transmitted premutations do not expand to full mutations). As the smallest allele to date reported to have expanded to a full mutation in a single generation is 56 repeats [23], it is appropriate also to offer prenatal diagnosis to women with intermediate alleles of 55 repeats or greater to allow for variations in sizing accuracy between different centres, although local policy will often determine the exact criteria used.

As with any prenatal test, it is important to exclude the possibility of maternal cell contamination of the fetal sample, which can be done with a variety of methods usually involving microsatellite analysis of the prenatal sample alongside a maternal blood sample. This is of particular importance for trinucleotide repeat expansions owing to the potential large size difference between normal and expanded alleles, and consequent possibility of preferential PCR amplification of the normal allele in even a tiny amount of contaminating maternal DNA.

If Southern blot analysis is required, the prenatal sample must be of sufficient size and quality to give a satisfactory yield of DNA to avoid the need for culturing and, hence, a long delay to the procedure; this means that chorionic villus samples are preferable to amniotic fluid, depending upon the stage of pregnancy. Commercial kits, if used instead of Southern blotting, must initially have been fully validated on prenatal samples.

**Figure 6:**
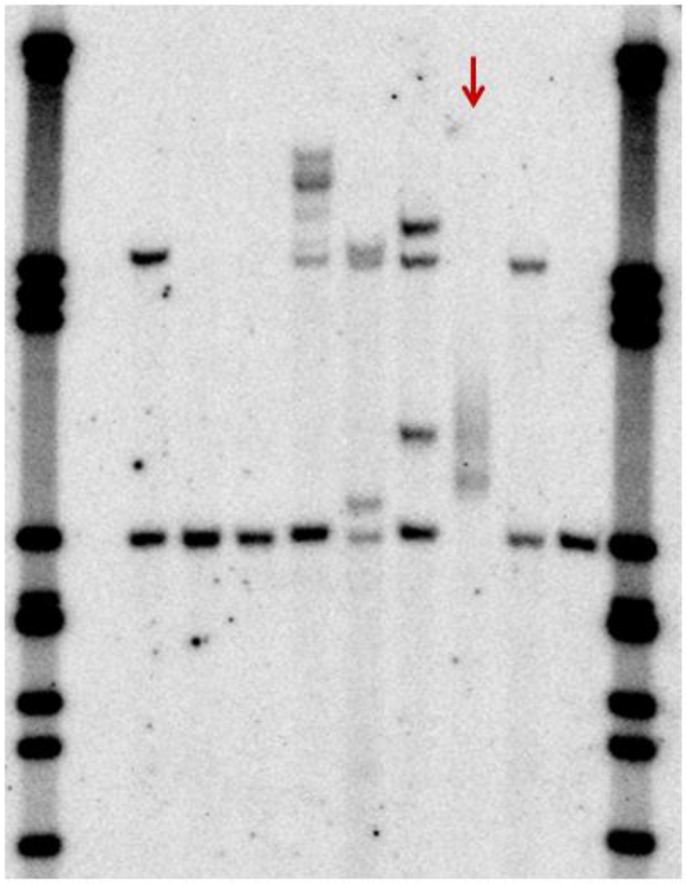
Southern blot showing male with unmethylated mosaic expansion (arrowed). The expansion size ranges from approximately 0.32–0.87 kb above normal size.

There are further considerations for the interpretation of prenatal results for Fragile X, principally that the methylation pattern observed in blood samples is not usually established in chorionic villus or amniotic fluid cells; hence, any expansion observed may need to be classified by size alone, which poses a challenge for any borderline pre/full mutation expansions identified. Although even full mutations are usually unmethylated in prenatal cells, the somatic mosaicism characteristic of a full mutation may be preserved, and this may aid in the interpretation (see Figure 6 for example of an unmethylated full mutation “smear” on a Southern blot).

In rare cases in which a clear prenatal diagnosis result is not provided by PCR, Southern blot or commercial kits, linkage analysis using microsatellites (if informative) may be useful. The three most informative microsatellite loci, *DXS548*, *FRAXAC1* and *FRAXAC2* flank the *FMR1* gene, have a high heterozygosity and show negligible recombination with the *FRAXA* locus. They have also proved useful in studies of the population genetics of Fragile X (see Section 3 above). Pre-implantation genetic diagnosis (PIGD) has been explored, but owing to the difficulty in detecting expanded alleles by PCR, a linkage-based approach may be more effective for this procedure. Another potential problem with PIGD is the reduced fertility or ovarian insufficiency in a proportion of female premutation carriers (see Section 7 below).

## 7. Testing for Other *FMR1*-Related Disorders

*FMR1* premutations have been found in up to 10% of cases of primary ovarian insufficiency (POI; also known as premature ovarian failure (POF) or secondary amenorrhoea, and defined as menopause before age 40). It is thought that ~24% of female premutation carriers will experience POI [43], although the risk is dependent on the size of the expansion in non-linear fashion [44] with medium-sized premutations (70–90 repeats) conferring the greatest risk. Repeats in the normal or intermediate range are not risk factors for POI or early menopause [45,46]. In the general population, women who go through menopause before 40 years are at approximately five-fold increased risk of carrying an *FMR1* premutation [47], while women with a premutation experience menopause on average five years earlier than those with normal alleles or a full mutation [48].

Referrals commonly request *FMR1* testing for patients with POI, but karyotyping is also still an appropriate first-line test: X; autosome translocations and X chromosome deletions have been detected in 2%–5% of POI cases. If the karyotype test is negative, then testing for an *FMR1* premutation should be considered; local policy will determine if this is triggered automatically or only done upon request.

Premutations have also been found in men over 50 with a progressive ataxia and tremor syndrome (Fragile X-associated tremor-ataxia syndrome; FXTAS). In this case, the risk shows a linear correlation with repeat size and an age-related penetrance rising from 17% at age 50 to 75% at age 80 or over [49]. Again, referrals for *FMR1* testing may be received from patients with a late-onset neurological phenotype, but it is less appropriate to initiate testing without a specific request as the typical symptoms show more overlap with other age-related conditions.

If a premutation is identified during routine Fragile X diagnostic testing or during cascade screening in Fragile X families, the risk of POI and/or FXTAS may need to be highlighted (depending upon the age of the patient) in addition to the implications related to Fragile X syndrome.

## 8. Interpretation and Reporting

The combination of methods used, as detailed above, should make interpretation and reporting straightforward for all but exceptional cases. All males with one allele and all females with two alleles in the normal range can safely be reported as normal on the assumption that mosaicism for a normal and a pre- or full mutation allele is extremely rare or non-existent, probably a safe assumption given that mosaics are thought to be due to reversion in size from a full mutation downwards and/or positive selection for a small pool of premutation-carrying cells among a population of predominantly full mutation-carrying cells. In females with only one allele detected by PCR, the Amplidex^TM^ kit can often determine homozygosity by dosage relative to a control autosomal allele, but Southern blotting may also be used to confirm this. The size of expanded alleles can be estimated with reasonable precision if detected by PCR, but only approximately by Southern blot using a standard size ladder for comparison. The precise repeat size is relatively unimportant for alleles clearly within the normal range or clearly within the full mutation range, but intermediate and premutation alleles as well as any borderline alleles are likely to require accurate sizing to enable a clear diagnosis.

The most difficult interpretations are likely to involve cases where: (i) methylation status is critical; (ii) mosaicism is detected; or (iii) where an allele of “borderline” size is identified, particularly at the boundary of intermediate and premutation size classes.

As methylation is not often established in chorionic villus or amniotic fluid samples referred for prenatal diagnosis, classification into full or pre-mutation needs to be done primarily by size estimation; for borderline cases (~200 repeats or ~0.6 kb above normal size), any evidence of extensive somatic mosaicism would push the judgement towards a full mutation. A cautionary rider stating that size/methylation status in a chorionic villus or amniotic fluid sample may not reflect that of the fetus can be added on reports; nevertheless, in our experience there is a good concordance between prenatal diagnosis results obtained from chorionic villi and follow-up tests on the newborn child when these have been carried out. In mosaic cases (where a pre- and a full mutation are detected) the approximate percentage of unmethylated premutation versus methylated full mutation detected may have some predictive value for the adult phenotype; this would need to be estimated either from signal strength on a Southern blot image or from a specialist methylation commercial kit, as PCR dosage would be subject to preferential amplification of smaller alleles. Additional information on methylation status may be gained from new techniques utilizing methylation-specific melt curve analysis [50,51] although this may add significant extra costs to the testing regime.

The reporting of intermediate alleles presents a particular challenge as the boundaries between normal, intermediate and premutation are to some extent subjective and difficult to define accurately. In the range 41–60 repeats, the likely stability of an *FMR1* allele has proven hard to predict with certainty as it depends upon other factors such as interspersion pattern and flanking haplotype; hence, previous publications have used different parameters for the intermediate range, making it difficult to compare studies of prevalence of intermediate and premutation alleles. As discussed above (Section 2 and Table 1) the lower size limit of premutations is now usually quoted as either 55 or 59 repeats: each of these is subject to possible error, and thus which one to use will depend upon how one defines a premutation, for what purpose the definition is to be used, and what proportion of type I versus type II errors (i.e., false positives versus false negatives) is considered acceptable. Many research-based publications now use 55 repeats or more as the definition of a premutation, which is designed to include most alleles that show any risk of instability, however minor. In our view, for diagnostic purposes in order to avoid creating too many false positives and, hence, unnecessary anxiety, 59 repeats is a more realistic and practical lower limit for the premutation allelic state; this definition would encompass only those alleles thought highly likely to show progressive and accelerating expansion in each succeeding generation, while those subject to occasional minor increments of 1–2 repeats are classified as intermediate.

To alleviate type II errors and allow for rare cases of pronounced instability within the intermediate range, it is necessary to state the possibility of instability and to recommend family follow-up to evaluate the stability of the allele in the family. A further consideration is the standard error of size estimates from PCR studies, likely to be +/−1 or +/−2 repeats in most laboratories for intermediate-sized alleles; hence, when considering whether to offer prenatal diagnosis, the cut-off may need to be conservative (e.g., 55 repeats) to reflect this uncertainty. Information on AGG interspersion pattern, if available, could aid considerably the reporting and genetics counselling of intermediate alleles.

Alleles clearly within the full mutation size range or clearly hypermethylated can be reported as consistent with a diagnosis of Fragile X syndrome in male patients, but in females where only a proportion of those with a full mutation show any symptoms, a more cautious interpretation may be warranted and alternative diagnoses not ruled out.

## 9. Future Directions and Conclusions

### 9.1. Diagnosis

It is likely that, in the future, universal diagnostic commercial kits will become both cheaper and more efficient at classifying and sizing all *FMR1* alleles, and also that third-generation sequencing methodologies will enable accurate sequencing through large repeat tracts; either approach may therefore become commercially viable as a first-line testing procedure, thus rendering Southern blotting increasingly obsolete for routine Fragile X diagnosis. Meanwhile, current next-generation sequencing platforms (panels of intellectual disability genes or clinical exome sequencing) could identify patients with point mutations and deletions in *FMR1*, which may have been missed by current techniques testing only the CGG repeat size, thus further refining the estimates of Fragile X prevalence.

### 9.2. Population Screening

Some experimental population screens have already been carried out, on both newborn populations [52,53] and on antenatal populations [54,55]. Population screening has the potential to improve the detection rate of Fragile X patients and identify affected families earlier than the current testing strategies, which could potentially reduce the burden on health resources both by preventing future Fragile X pregnancies in positive cases and by redirecting genetic testing towards other intellectual disability genes in negative cases. The disadvantage, as ever with large-scale screens, is the inevitable detection of additional unwanted information in the form of carriers of premutations and intermediate alleles with consequent problems for genetics counselling; hence, it is likely that future screening programmes will be designed to focus solely on full mutations, whether by detecting hypermethylation of the *FMR1* gene [50] or the presence/absence of FMRP protein [56].

### 9.3. Therapy

The continued elucidation of the role of FMRP in the pathogenesis of Fragile X syndrome opens up more potential therapeutic options, particularly if combined with early detection of the condition as a result of newborn screening. Trials of drugs that target mGluR5 showed initial promise in model systems but clinical trials were abandoned due to lack of efficacy. Knockout mouse models exhibit downregulation of the gamma-aminobutyric acid (GABA) system and thus GABA agonists are the focus of current clinical trials [57]. Reactivation of the methylated *FMR1* gene with the demethylating agent 5-azadeoxycytidine is being tested in in vitro models and may provide a potential therapeutic approach [58].

### 9.4. Concluding Remarks

In the 25 years since the elucidation of the molecular basis of Fragile X syndrome, it has transformed from—in the words of Professor Patricia Jacobs—“the most fascinating and puzzling problem in human genetics” to a routine diagnostic procedure forming one of the most common outputs for health services around the world. Even now, its genetic basis remains unique in detail and provides particular challenges shared by no other condition. Awareness of Fragile X syndrome and its implications has improved considerably among both clinical and scientific healthcare professionals, though not necessarily among the public at large; nevertheless, the information available to Fragile X families and their options for reproductive choices and therapy are greatly enhanced. The challenges in the future are to provide adequate financial and counselling support for patients and their families in spite of a stringent economic climate and to fund the most appropriate therapies targeted at the particular needs of Fragile X patients throughout their lives.

## Figures and Tables

**Table 1:** Allele category definitions in common usage.

Allele	UK		USA	
	Repeat size	Methylation status	Repeat size	Methylation status
Normal	0–45	Regular	0–44	Regular
Intermediate	46–58	Regular	45–54	Regular
Premutation	59–200 *	Regular	55–200 *	Regular
Full mutation	>200 *	Hypermethylated *	>200 *	Hypermethylated *

* Current techniques cannot give a precise sizing at the level of hundreds of repeats and there is an overlap of repeat sizes which may be methylated or unmethylated, so the distinction between premutation and full mutation is usually made by combining observations of size, methylation status and degree of somatic mosaicism; hence, there are examples of unmethylated full mutations as well as of premutations estimated at >200 repeats. “Unmethylated” is hitherto used to refer to the regular state of DNA in which CpG motifs are not methylated and therefore digestible by methylation-sensitive restriction enzymes.
